# ER Stress in COVID-19 and Parkinson’s Disease: In Vitro and In Silico Evidences

**DOI:** 10.3390/brainsci12040507

**Published:** 2022-04-16

**Authors:** Zahara L. Chaudhry, Mahmoud Gamal, Ingrid Ferhati, Mohamad Warda, Bushra Y. Ahmed

**Affiliations:** 1 Institute of Biomedical & Environmental Science and Technology, School of Life Sciences, Faculty of Creative Arts, Technologies & Science, University Square, University of Bedfordshire, Luton LU1 3JU, UK; zahara.chaudhry@beds.ac.uk (Z.L.C.); Ingrid.Ferhati1@beds.ac.uk (I.F.); 2 Department of Biochemistry and Molecular Biology, Faculty of Veterinary Medicine, Cairo University, Giza 12211, Egypt; mahmoud.gamal@cu.edu.eg (M.G.); maawarda@cu.edu.eg (M.W.)

**Keywords:** Parkinson’s disease, SARS-CoV-2, caspase, endoplasmic reticulum (ER) stress, PKR-like ER kinase (PERK), 6OHDA, oxidative stress

## Abstract

The outbreak of COVID-19 caused by severe acute respiratory syndrome coronavirus 2 (SARS-CoV-2) signifies a serious worldwide concern to public health. Both transcriptome and proteome of SARS-CoV-2-infected cells synergize the progression of infection in host, which may exacerbate symptoms and/or progression of other chronic diseases such as Parkinson’s disease (PD). Oxidative stress is a well-known cause of endoplasmic reticulum (ER) stress observed in both SARS-CoV-2 and PD. In the current study, we aimed to explore the influence of PKR-like ER kinase (PERK) stress pathway under SARS-CoV-2-mediated infection and in human cell model of PD. Furthermore, we investigated whether they are interconnected and if the ER stress inhibitors could inhibit cell death and provide cellular protection. To achieve this aim, we have incorporated in silico analysis obtained from gene set enrichment analysis (GSEA), a literature review and laboratory data. The neurotoxin, 6-hydroxy dopamine (6OHDA), was used to mimic the biochemical and neuropathological characteristics of PD by inducing oxidative stress in dopamine-containing neurons differentiated from ReNVM cell line (dDCNs). Furthermore, we explored if ER stress influences activation of caspases-2, -4 and -8 in SARS-CoV-2 and in stressed dDCNs. Our laboratory data using Western blot, immunocytochemistry and 3-(4,5-dimethylthiazolyl-2)-2,5-diphenyltetrazolium bromide (MTT) analyses indicated that 6OHDA-induced toxicity triggered activation of caspases-2, -4 and -8 in dDCNs. Under SARS-CoV-2 infection of different cell types, GSEA revealed cell-specific sensitivities to oxidative and ER stresses. Cardiomyocytes and type II alveolar epithelial-like cells were more vulnerable to oxidative stress than neural cells. On the other side, only cardiomyocytes activated the unfolded protein response, however, the PERK pathway was operative in both cardiomyocytes and neural cells. In addition, caspase-4 activation by a SARS-CoV-2 was observed via in silico analyses. These results demonstrate that the ER stress pathway under oxidative stress in SARS-CoV-2 and PD are interconnected using diverse pathways. Furthermore, our results using the ER stress inhibitor and caspase specific inhibitors provided cellular protection suggesting that the use of specific inhibitors can provide effective therapeutic approaches for the treatment of COVID-19 and PD.

## 1. Introduction

The ongoing COVID-19 pandemic is a global threat to human health. One of the reasons for serious pathogenicity manifested by severe acute respiratory syndrome coronavirus 2 (SARS-CoV-2) is potentially due to oxidative stress, leading to subcellular organelles defects including endoplasmic reticulum (ER) [1,2,3,4] and mitochondrion [5]. The ER, besides being the site for cellular homeostasis with Ca^2+^ ions cache, lipid and protein synthesis [6], it is responsible for protein maturation and the disposal of misfolded protein aggregates [7,8]. Subsequently, the correctly folded mature proteins move from the ER to the Golgi body where they are further post-translationally modified and released to their target destinations. However, elevated levels of accumulated misfolded proteins due to inflammation, oxidative stress, genetic mutation, glucose deprivation, disruption of disulfide bond formation, cytosolic calcium imbalance, toxin exposure and viral infection exert profound impact on ER status [9,10,11]. In normal circumstances, incorrectly folded proteins are sensed by the ER-associated degradation (ERAD) pathway and are subsequently degraded by the ubiquitin protease system (UPS) [7,8,12,13,14]. The ER-embedded transmembrane proteins such as PKR-like ER kinase (PERK) is a well-known sensor for ER stress. It plays an important role in maintaining cellular homeostasis during ER stress [1,15]. Furthermore, the exposed hydrophobic regions of the misfolded protein form toxic aggregates such as fibrils and trigger ER stress [16,17,18]. The insoluble fibrils co-precipitate and polymerize into lewy bodies, a key attribute found in Parkinson’s disease (PD) patients [18,19,20]; it is also seen frequently in patients with anosmia [21], a coherent clinical sign observed in COVID-19 patients [22]. Moreover, the activation of ER stress marker GRP78 [23,24] and PERK pathway [1] along with their increased serum levels following SARS-CoV-2-mediated stress has also been recognized in COVID-19 patients.

A work by Colla et al. [25] found that the ER-accumulated α-synuclein oligomers activate ER stress in transgenic mouse model. Western blot analysis of the midbrain from the transgenic mouse model revealed high levels of aggregated α-synuclein in mitochondrial and ER microsomal fractions with increased fragmentation of Golgi body. On the other hand, treatment with salubrinal, a potent PERK inhibitor resolved these α-synucleins aggregations with the minimization of Golgi body fragmentation in the same transgenic model. This clearly demonstrates that aggregated α-synuclein provides a signal to trigger ER stress.

The neurotoxins 6-hydroxydopamine (6OHDA) and 1-Methyl 4-Phenylpyridinium (MPP^+^) have been shown to mimic the key attributes of PD such as lewy body formation, mitochondrial dysfunction and reactive oxygen species (ROS) production, hence are commonly used neurotoxins to study in vivo [26,27,28] and in vitro [29] PD models.

A study on caspases-2,-3,-4 and -9 activations using caspase-specific colorimetric substrates such as ac-VDVAD-fmk, ac-DEVD-fmk, ac-LEVD-fmk and ac-LEDH-fmk, along with immunofluorescence analysis using Fura-2AM, demonstrated an increased calcium levels in the ER and mitochondria of thapsigargin and MPP^+^-treated cells. These results collectively demonstrated that MPP^+^ triggers calcium leakage from the ER into the mitochondria provoking activation of caspases-2,-3,-4 and -9 with consequent death of neuronal cells [30].

We have recently shown that 6OHDA-induced oxidative stress triggers the activation of NFκB classical pathway, which plays a vital role in caspase-mediated apoptotic death in dopamine-containing neurons differentiated from ReNVM cell line (dDCNs). Furthermore, we have demonstrated the similarities in inflammatory pathways between COVID-19 and PD following oxidative stress and their links with NFκB activation [26]. Here, we study whether the ER stress pathway under oxidative stress in SARS-CoV-2 and PD are interconnected and if the potential use of ER stress inhibitors favors cellular protection. To initiate this aim, the laboratory study and bioinformatics data along with literature review were used to investigate the impact of PERK-ER stress pathway under SARS-CoV-2-mediated infection and in dDCNs following 6OHDA-induced oxidative stress [26,31]. In addition, we investigated if ER stress influences activation of caspases-2, -4 and -8 in 6OHDA-treated dDCNs.

Oxidative stress, a critical pathway in PD development and in the pathogenesis of progressive supranuclear palsy [32,33], a typical parkinsonian syndrome was assessed by scoring gene expression profile in SARS-CoV-2 infected cells. Furthermore, ER stress, another important pathway in developing PD was examined in SARS-CoV-2 infected cells by evaluating the transcriptomic signature of the unfolded protein response as represented by a gene set called hallmark-unfolded protein response that was obtained from the molecular signatures database [34]. Additionally, under the umbrella of the ER stress, the transcriptomic signature of PERK activation that plays a critical role in the development of PD was assessed in SARS-CoV-2 infection by gene set obtained from Reactome database (Reactome-PERK regulates gene expression). As PERK can activate the antioxidant transcription factor nuclear erythroid 2-related factor 2 (Nrf2), the transcriptomic signature of the Nrf2 was assessed using BioCarta-ARENRF2 pathway [35].

## 2. Materials and Methods

### 2.1. In Silico Analyses

#### 2.1.1. Gene Set Enrichment Analysis (GSEA) of ER Stress and Oxidative Stress in SARS-CoV-2 Infected Cells

To examine ER stress and oxidative stress in response to COVID-19 infection, we re-analyzed three gene expression series publicly available on the gene expression omnibus (GEO) [36]. The gene expression series were GSE150392, GSE153277, and GSE157852, representing three types of the induced pluripotent stem cells (iPSC): iPSC-cardiomyocytes, iPSC-type II alveolar epithelial-like cells and iPSC-neural cells, respectively [37,38,39,40]. Raw or normalized counts file of each gene expression series were downloaded from the GEO and were loaded on RStudio v1.4.1717 [41] using R programming language v4.1.0 [42]. The reads per million reads (RPM) were calculated to normalize raw counts files.

Text file format for expression datasets were prepared from the RPM-normalized files according to GSEA instructions and loaded on GSEA v4.1.0 software along with their respective phenotypes files [43,44]. GSEA was run using gene sets obtained from the molecular signatures database (MSigDB) [34]. The used gene sets were hallmark-unfolded protein response [34] and Reactome-PERK regulates gene expression (R-HSA-381042) were used to examine ER stress specifically in the PERK pathway. While, Reactome-oxidative stress induced senescence (R-HSA-2559580) and BioCarta-ARENRF2 pathway [35] were used to evaluate oxidative stress. The default GSEA parameters, except for the permutation type, were used to adjust gene set.

#### 2.1.2. Caspase-4 and SARS-CoV-2 Proteases

In order to test if human caspase-4 is a target of SARS-CoV-2 proteases, we used NetCorona-1.0 an online server that searches for 3C-like proteinase cleavage sites and gives a score for the accuracy of these predictions [45]. Briefly, we obtained the caspase-4 protein sequence in FASTA format from the NCBI protein database accession number (P49662.1) and used NetCorona-1.0 to assign scores (from 0 to 1) for each glutamine residue in caspase-4. Scores received higher than 0.5 (NetCorona-1.0 threshold) were predicted as actual cleavage sites.

### 2.2. Laboratory-Based Analyses

#### 2.2.1. Chemicals and Antibodies

Reagents: ReNVM NSC maintenance medium (SCM005; Millipore, Watford, UK).

Primary and secondary antibodies: Anti-tyrosine hydroxylase (TH), was purchased from Millipore, Hertfordshire, UK, anti-caspase-2 (ab7979), anti-caspase-4 (ab97318) and anti-caspase-8 (ab52183) were purchased from Abcam, Cambridge, UK. Goat anti-rabbit IgG-HRP, goat anti-rabbit IgG-rhodamine, donkey anti-mouse IgG-FITC were purchased from Millipore, Hertfordshire, UK.

Inhibitors: Salubrinal (Bergheimer, Heidelberg, Germany), caspase-2 inhibitor zVDVADfmk (218744); caspase-4 inhibitor zLEVDfmk (218755) and caspase-8 inhibitor zIETDfmk (218759) were obtained from Merck Chemicals, Nottingham, UK; the universal caspase inhibitor zVADfmk (G7351) was purchased from Promega, Southampton, UK.

#### 2.2.2. Cell Culture and Treatments

ReNVM cells (Millipore, Watford, UK) were differentiated into dopaminergic neurons (dDCNs) and were cultured for Western blot (WB), immunocytochemical and 3-(4,5-dimethylthiazolyl-2)-2,5-diphenyltetrazolium bromide (MTT) analyses as described previously [26]. The use of the dopamine marker TH, a common practice in our group was used to ensure that ReNVM had differentiated into DCNs (dDCNs). These cells were treated with 100 µM 6OHDA for 2 h to induce stress in all experiments, the media was replaced with fresh media, and dDCNs were left in the incubator for overnight at 37 °C and collected after 24 h; as previously described [26,31], these cells were labelled as 6OHDA-treated dDCNs. The untreated control group went through media change only.

To determine the influence of PERK pathway on specific caspases, control and 6OHDA-treated dDCNs were treated for 2 h with 30 µM salubrinal in the presence of different combinations of specific caspase inhibitors such as 20 µM zVDVADfmk, 20 µM zLEVDfmk and 80 µM zIETDfmk for caspases-2,-4 and -8, respectively, after which, media was removed and replaced with fresh media and the cells were left to recover overnight. The optimal condition for salubrinal dose of 30 µM was established by incubating dDCNs with or without 100 µM 6OHDA for 2 h followed by treatment with different concentration of (30 µM–120 µM) salubrinal (data not shown). Immunocytochemical, WB, cell viability and statistical analyses were performed as described below.

#### 2.2.3. Western Blot Analysis

Control, 6OHDA-treated, salubrinal-treated, and 6OHDA+salubrinal-treated dDCNs were used for WB analyses as previously described [26]. Briefly, dDCNs were grown and treated with 30 µM salubrinal, 100 µM 6OHDA, or a mixture of 100 µM 6OHDA and 30 µM salubrinal for 2 h, after which, the old media was removed and replaced with fresh media and cells were left to recover overnight at 37 °C in CO_2_ incubator. Cells were lysed and 50 µg of protein was loaded on a 12% gel prior to SDS PAGE, followed by immunoblotting on a PVDF membrane.

The primary antibodies, anti-caspase-2 (1:2500), anti-caspase-4 (1:300) and anti-caspase-8 (1:1000), and the secondary antibody goat-anti-rabbit HRP (1:1000) were used.

After washing the membranes with PBST, an enhanced chemiluminescence system (ECL) was employed and immunoblots bands were quantified with a densitometer scanner followed by stripping and re-probing the membranes with the housekeeping gene GAPDH. Three to five independent experiments were performed for each treatment.

#### 2.2.4. Detection and Statistical Analysis of Western Blot Data

Band analysis was carried out using a densitometer (GS800, Bio-Rad, Hertfordshire, UK). Blots were scanned using GS800 scanner, the band lanes and bands were detected using Quantity One software at default settings, and the raw densitometric intensity values were obtained as previously described [26]. The housekeeping protein GAPDH was used as a loading control and the densitometric values for all samples were normalized against GAPDH to determine the densitometric ratio prior to carrying out statistical analysis (Student’s *t*-test, *p* < 0.05, ANOVA, *p* < 0.05). All of the results are presented as relative percentage compared with control. Between 3 and 5 independent experiments were performed for each treatment and values are indicated as means ± standard error means (M ± SEM) and analyzed with SPSS software (IBM SPSS v.23.0, Chicago, IL, USA). Comparisons between different groups were determined using one-way analysis of variance (ANOVA) followed by post-hoc multiple comparison tests. Statistical significance was set at *p* < 0.05. Table of values and statistical analysis can be found in the Supplementary Materials; the relevant number of experiments performed is mentioned in the figure legends.

#### 2.2.5. Immunocytochemistry

Immunocytochemical analysis was performed to investigate if salubrinal could inhibit expression of caspases-2, -4 and -8 in 6OHDA- treated dDCNs. Control and 6OHDA-treated dDCNs were treated for 2 h with 30 µM salubrinal and specific caspase inhibitors for caspases-2,-4 and -8 as mentioned above (Section 2.2.2), after which, old media was replaced with fresh media and the cells were left to recover overnight. The standard immunocytochemical procedure as described previously was followed [26]. Briefly, cells were fixed with 4% paraformaldehyde, washed with PBS and were treated with Triton x-100 followed by blocking with 10% goat serum for 40 min prior to overnight incubation with primary antibodies anti- TH (1:1500), anti-caspase-2 (1:2000), anti-caspase-4 (1:4000), anti-caspase-8 (1:2000) at 4 °C. The cells were incubated with secondary antibody donkey-anti-mouse IgG FITC (1:500) or goat-anti-rabbit IgG rhodamine (1:2500) for 2 h at room temperature. Cells were mounted using vectorshield mounting medium and were viewed under a Meiji fluorescent microscope.

#### 2.2.6. MTT Assay

The MTT (Cambridge Bioscience, UK) assay was performed to measure survival of dDCNs following different treatments in control and 6OHDA-treated dDCNs as described previously [26]. Briefly, dDCNs were grown in 96 well plates and were left until they were 80% confluent and then were then treated with different combinations of salubrinal, zVDVADfmk, zIETDfmk and zLEVDfmk (see figures for details) with or without 6OHDA for 2 h, after which old media was replaced with fresh media and cells were left to recover overnight as described above. The following day, an MTT assay was performed, a microplate reader and the program Stingray was used to determine the readings of samples at 570 nm wavelength. The readings were normalized prior to statistical analysis at *p* < 0.05 using ANOVA and Student’s *t-test*. Three independent experiments were conducted each for untreated, 6OHDA-treated and inhibitor(s)-treated dDCNs.

## 3. Results

### 3.1. In Silico Results

#### 3.1.1. Oxidative Stress Gene Sets Enrichment Analysis Results

**Figure 1:**
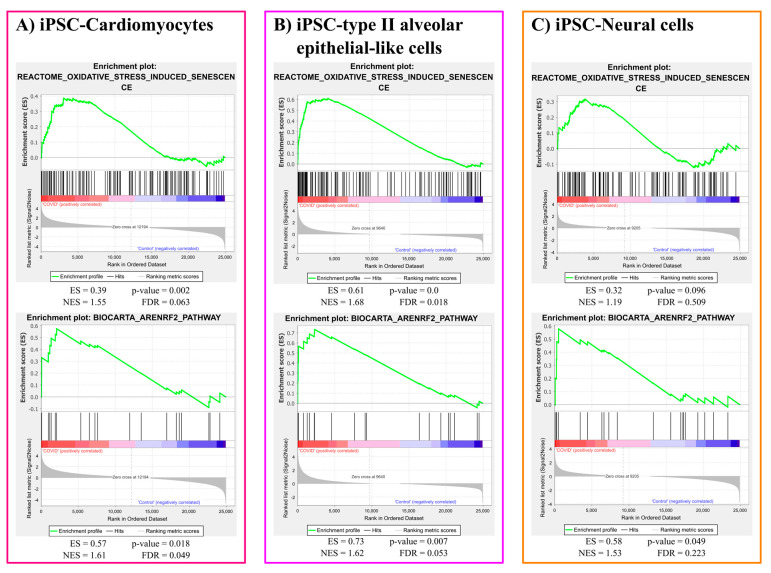
Oxidative stress gene sets enrichment analysis results. ES: enrichment score, NES: normalized enrichment score, FDR: false discovery rate.

Oxidative stress induced senescence gene set was significantly enriched in response to COVID-19 infection in iPSC-Cardiomyocytes (*p*-value = 0.002) and iPSC-type II alveolar epithelial-like cells (*p*-value < 0.001). However, the same gene set was not significantly (*p*-value = 0.096) enriched in the case of iPSC-neural cells infected with COVID-19. However, Nrf2 responsive gene set was robustly enriched due to COVID-19 infection in the three cell types: iPSC-Cardiomyocytes (*p*-value = 0.018), iPSC-type II alveolar epithelial-like cells (*p*-value = 0.007), and iPSC-neural cells (*p*-value = 0.049). The plots for oxidative stress gene sets enrichment results are represented in Figure 1.

#### 3.1.2. ER Stress Gene Sets Enrichment Analysis

**Figure 2:**
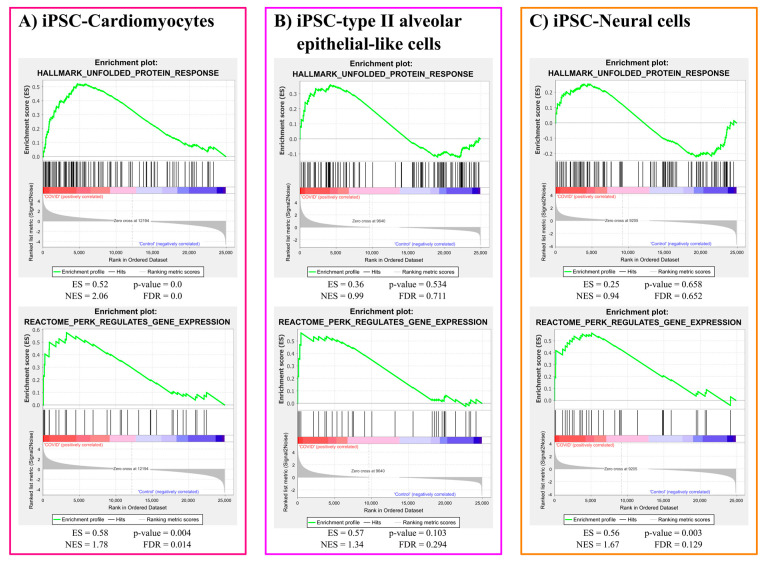
ER stress gene sets enrichment analysis results. ES: enrichment score, NES: normalised enrichment score, FDR: false discovery rate.

The unfolded protein response (UPR) gene set was significantly activated in iPSC-cardiomyocytes in response to COVID-19 infection (*p*-value < 0.001). However, the same gene set did not react significantly to COVID-19 infection in iPSC-type II alveolar epithelial-like cells (*p*-value = 0.534) and iPSC-neural cells (*p*-value = 0.658). Furthermore, the gene set expressed in response to PERK activation, a downstream arm of the UPR, was significantly enriched due to COVID-19 infection in iPSC-cardiomyocytes (*p*-value = 0.004) and iPSC-neural cells (*p*-value = 0.003). However, COVID-19 infected iPSC-type II alveolar epithelial-like cells showed insignificant (*p*-value = 0.103) enrichment in the PERK activated gene set. The plots for ER stress gene sets enrichment results are represented in Figure 2.

#### 3.1.3. 3CL Protease Is Predicted to Cleave and Activate Caspase-4

From the amino acid sequence of caspase-4, we found that it has 14 glutamine residues that represent candidate cleavage sites for 3CL protease. Using NetCorona-1.0, our results show that caspase-4 could be cleaved by the 3 CL protease of SARS-CoVs at Gln281 with a cleavage score of 0.630, which is higher than the NetCorona-1.0 server threshold (0.5) as represented in Table 1.

### 3.2. Laboratory-Based Results

#### 3.2.1. Salubrinal and zVADfmk Promote Survival of Stressed dDCNs

**Figure 3:**
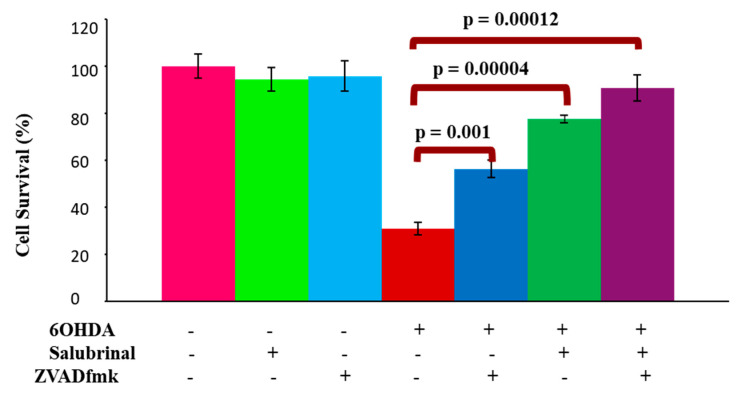
6OHDAinduced oxidative stress stimulates activation of PERK and caspases in dDCNs. A significant increase in dDCNs survival was observed in cells that had been treated with salubrinal+ zVADfmk following 6OHDA treatment (91% cell survival *p* < 0.01). The proportion of cells surviving was determined by MTT absorbance at 570 nm. Means of three experiments ± SEM is shown. A table of values and statistical analysis can be found in Supplementary Material S1.

To determine if 6OHDA-induced oxidative stress can trigger ER stress-mediated apoptotic death in dDCNs, the effect of the specific PERK inhibitor salubrinal on the viability of dDCNs was studied in control and 6OHDA-treated dDCNs. Salubrinal prevented apoptotic death in 6OHDA-treated dDCNs and promoted their survival (78% cell survival, *p* < 0.001) compared to 31% in 6OHDA-treated dDCNs. This indicates that oxidative stress activates the PERK pathway due to ER stress leading to death of dDCNs (Figure 3).

To confirm if ER stress is not the only pathway causing death to 6OHDA-treated cells, different concentrations of salubrinal were used (30 µM–120 µM). The results indicated that increasing the concentration of salubrinal did not completely inhibit death of 6OHDA-treated dDCNs (data not shown). These results suggest the involvement of another cell signaling pathway in cell death of 6OHDA-treated dDCNs as well.

The broad caspase inhibitor zVADfmk was used to investigate the involvement of caspase pathway and if suppression of UPR could prevent activation of caspases in 6OHDA-induced stressed dDCNs. In addition, the combination of zVADfmk and salubrinal was used to determine the synergistic effect of both inhibitors on cell survival following 6OHDA treatment. The combination of zVADfmk and salubrinal promoted pronounced cell survival of 6OHDA-treated dDCNs (91%) compared to the cell survival effects of zVADfmk (56%) or salubrinal (78%) per se. The additional survival of stressed dDCNs when using both inhibitors indicated that ER stress is not the only cell signaling pathway that is causing apoptotic death of dDCNs (Figure 3).

#### 3.2.2. 6OHDA-Induced Oxidative Stress Stimulates Caspase-2 Activation via the ER Stress Pathway in dDCNs

**Figure 4:**
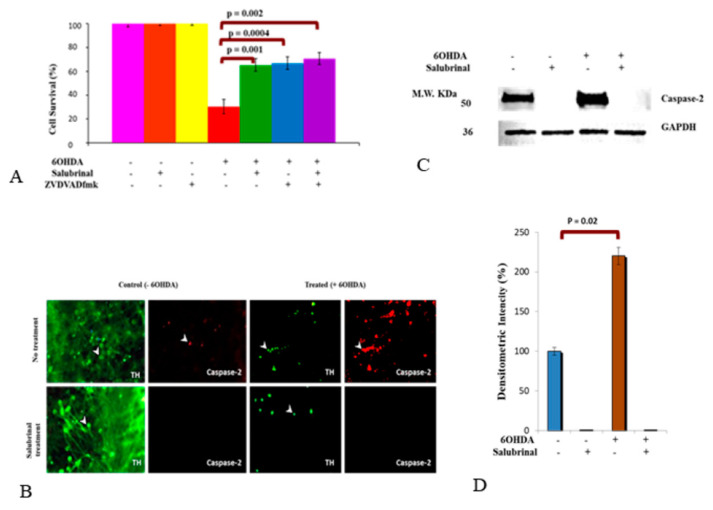
Activation of caspase-2 in ER stress pathway. (**A**) Control and 6OHDA-treated dDCNs were treated with zVDVADfmk and salubrinal. A 65% cell survival was observed in stressed dDCNs treated with salubrinal, whereas 67% cell survival was measured in zVDVADfmk-treated dDCNs after 6OHDA-induced stress. The combined effect of both inhibitors provided 71% dDCNs survival in 6OHDA+salubrinal+zVDVADfmk treated-dDCNs. The proportion of cells surviving was determined by MTT absorbance at 570nm. (**B**) Figure shows positive staining for TH in control and 6OHDA-treated dDCNs (green). A loss of dendrites was observed in TH positive dDCNs that had been exposed to 6OHDA. Caspase-2 was present in control and 6OHDA-treated TH positive dDCNs (red). There were more caspase-2 positive cells present in 6OHDA-treated dDCNs compared to untreated dDCNs. Caspase-2 was absent in salubrinal-treated and 6OHDA+salubrinal- treated dDCNs. This indicates that inhibiting the PERK pathway can suppress caspase-2 activity in dDCNs. The white arrow represents positive staining for cell bodies in dDCNs. (**C**) Illustrative example of GAPDH and caspase-2 in untreated, salubrinal-treated, 6OHDA-treated and salubrinal+6OHDA-treated dDCNs. (**D**) Quantitatively, a significant increase in the caspase-2 level was detected in 6OHDA-treated dDCNs compared to control dDCNs (*p* < 0.05). The absence of caspase-2 in salubrinal-treated and 6OHDA+salubrinal-treated dDCNs suggests that caspase-2 activity is dependent upon the PERK pathway. Means of five experiments ± SEM is shown. A table of values and statistical analysis can be found in Supplementary Material S2 and S3 for (**A**,**D**), respectively.

We have recently shown that 6OHDA-induced oxidative stress triggered caspase-2 activation in dDCNs via the NFκB classical pathway [26]. Here, we aimed to determine if the PERK pathway influences activity of caspase-2 in 6OHDA-induced stressed dDCNs. To achieve this aim, the impact of zVDVADfmk, a caspase-2 inhibitor and salubrinal were investigated either individually or together in 6OHDA-treated dDCNs. The results demonstrated that salubrinal or both salubrinal and zVDVADfmk provided similar levels of cell protection (65–71% compared to 30%) in 6OHDA-treated dDCNs, suggesting that 6OHDA-induced oxidative stress triggered PERK and caspase-2 activation via the same pathway in dDCNs (Figure 4A).

Furthermore, immunocytochemical analysis using TH and salubrinal was used. As expected, a reduction in number of dDCNs and dendrites were observed following 6OHDA-induced oxidative stress in dDCNs compared to control (Figure 4B; green, upper panel). Salubrinal treatment showed enhanced expression of TH in control dDCNs compared to 6OHDA-treated dDCNs (Figure 4B, lower panel). Enhanced expression of caspase-2 was observed in 6OHDA-treated dDCNs compared to the control (Figure 4B; red, upper panel), indicating that 6OHDA triggers caspase-2 activation in dDCNs. Caspase-2 was absent in dDCNs treated with salubrinal in both control and 6OHDA-treated dDCNs (Figure 4B; red, lower panel).

To determine the level of caspase-2 suppression by salubrinal in 6OHDA-treated dDCNs, WB analysis was performed. Results demonstrated absence of the caspase-2 in salubrinal-treated and 6OHDA+salubrinal-treated dDCNs, indicating that 6OHDA-induced oxidative stress triggered the activation of caspase-2 via the PERK pathway in dDCNs. This also indicates that caspase-2 activation can be suppressed by directly inhibiting caspase-2 or via inhibiting the PERK-ER stress pathway in dDCNs (Figure 4C,D).

#### 3.2.3. Activation of Caspase-4 in 6OHDA-Treated dDCNs

**Figure 5:**
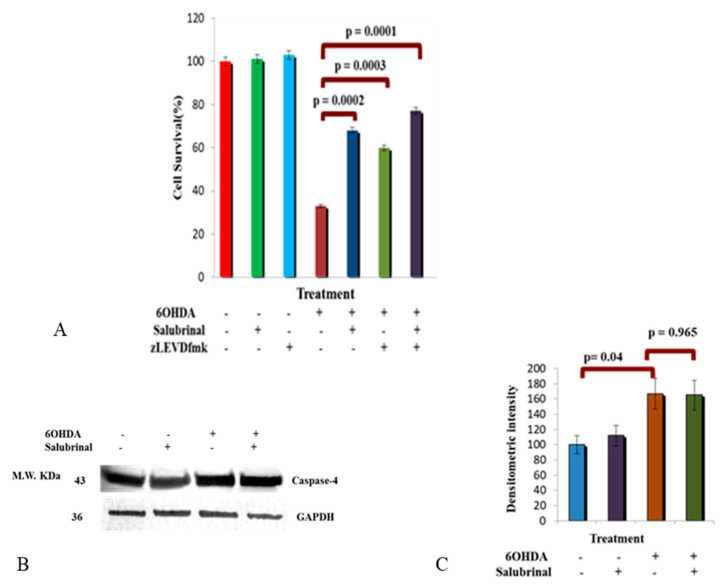
6OHDA triggered caspase-4 activity in dDCNs. (**A**) A 68% cell survival was determined in 6OHDA+ salubrinal-treated dDCNs, showing that 6OHDA triggered the PERK pathway. A 60% cell survival was measured in 6OHDA+zLEVDfmk-treated dDCNs, demonstrating 6OHDA-induced stress triggered caspase-4 activation in dDCNs. A further 77% survival of 6OHDA+salubrinal + zLEVDfmk-treated dDCNs, indicates that the PERK pathway does not influence caspase-4 activation in dDCNs. zLEVDfmk in combination with salubrinal promoted further survival of 6OHDA-treated dDCNs, indicating an additional benefit of targeting the ER stress pathway and caspase-4. The proportion of cells surviving was determined by MTT absorbance at 570 nm. Means of three experiments ± SEM is shown (**B**). Illustrative example of GAPDH and caspase-4 in control, salubrinal, 6OHDA and salubrinal+6OHDA-treated dDCNs (**C**). Quantitatively, a 67% increase in caspase-4 was detected in 6OHDA-treated dDCNs compared to control dDCNs (*p* < 0.05). Salubrinal did not show any effect on the amount of caspase-4 in salubrinal-treated and 6OHDA+salubrinal-treated dDCNs (*p* > 0.05). Means of five experiments ± SEM is shown. A table of values and statistical analysis can be found in Supplementary Materials S4 and S5 for (**A**,**C**), respectively.

Here, we aimed to determine if caspase-4 activity is influenced by the PERK-ER stress pathway in 6OHDA-treated dDCNs via the use of salubrinal and zLEVDfmk inhibitors (Figure 5). The combination of salubrinal and caspase-4 inhibitor provided a further increase in cell survival, showing that suppressing the caspase-4 pathway in addition to the PERK pathway had an added effect on cell survival, indicating the presence of at least two distinct pathways that are contributing to death of stressed dDCNs (Figure 5A). It also suggests that caspase-4 does not follow the PERK pathway and perhaps is activated by another pathway, which results in the death of dDCNs. WB analysis was performed to confirm the influence of salubrinal on caspase-4 activity following 6OHDA-induced oxidative stress in dDCNs. Results showed the presence of caspase-4 in control, salubrinal-treated, 6OHDA-treated and in 6OHDA+salubrinal-treated dDCNs. Additionally, an increased amount of caspase-4 was detected in 6OHDA-treated dDCNs compared to control dDCNs.

The ineffectiveness of salubrinal against the activation of caspase-4 further supports the notion that caspase-4 is independent of ER-mediated death in 6OHDA-treated dDCNs. These results suggest that activation of caspase-4 following 6OHDA-induced toxicity did not follow the PERK pathway (Figure 5B,C).

#### 3.2.4. Caspase-8 Activation by 6OHDA Is Independent on ER Stress Pathway

**Figure 6:**
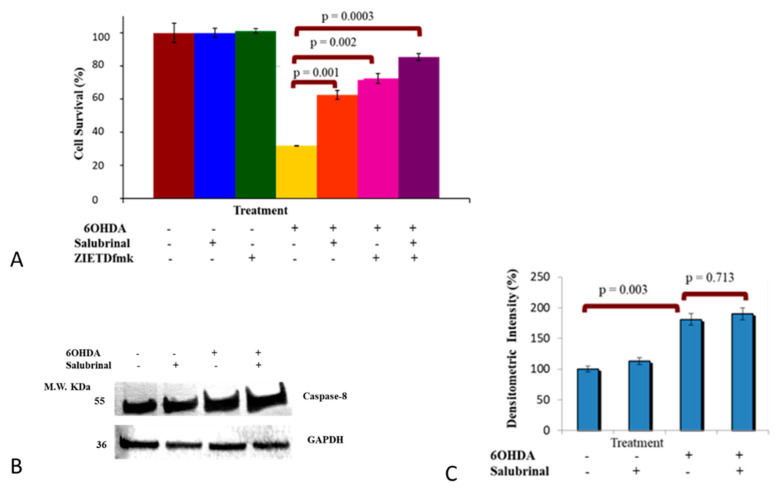
Salubrinal and zIETDfmk promote survival of 6OHDA-treated dDCNs (**A**) A 63% cell survival was determined in 6OHDA+ salubrinal-treated dDCNs, showing 6OHDA-induced stress has triggered the PERK pathway. A 72% cell survival was measured in 6OHDA+ zIETDfmk treated dDCNs, demonstrating 6OHDA triggered caspase-8 activation in dDCNs. A survival of 85% was seen in 6OHDA+ salubrinal + zIETDfmk-treated dDCNs, indicating that the PERK pathway does not influence caspase-8 activation in dDCNs. zIETDfmk in combination with salubrinal promoted further survival of 6OHDA-treated dDCNs, indicating an additional benefit of targeting ER stress pathway and caspase-8. The proportion of cells surviving was determined by MTT absorbance at 570 nm. Means of three experiments ± SEM is shown. (**B**) Illustrative example of GAPDH and active caspase-8 in control, salubrinal-treated, 6OHDA-treated and salubrinal+6OHDA-treated dDCNs (**C**). Quantitatively, an 81% increase in caspase-8 level was detected in 6OHDA-treated dDCNs compared to control dDCNs (100%) (*p* < 0.05). There was no effect of salubrinal treatment on caspase-8 level in salubrinal-treated and 6OHDA+salubrinal-treated dDCNs (*p* > 0.05). Means of three experiments ± SEM is shown. A table of values and statistical analysis can be found in Supplementary Materials S6 and S7 for (**A**,**C**), respectively.

We have recently shown that 6OHDA-induced stress enhances expression of caspase-8 in dDCNs with reference to the NFkB pathway [26]. To date, research regarding the association of caspase-8 with ER stress in dDCNs has not been investigated. Hence, it was of interest to determine if caspase-8 is active in the ER-mediated death pathway following 6OHDA-induced stress in dDCNs. The effect of zIETDfmk, a caspas-8 inhibitor and salubrinal either individually or in combination was studied. Results indicated that both caspase-8 and PERK are activated and provided cytoprotection following oxidative stress induced by 6OHDA, but they are following different pathways (Figure 6A). Furthermore, WB analysis confirmed that salubrinal did neither prevent nor reduce the amount of caspase-8 in 6OHDA-treated dDCNs, indicating that salubrinal does not influence caspase-8 activation. The ineffectiveness of salubrinal against activation of caspase-8 further supports the notion that caspase-8 is independent of the PERK pathway and does not follow ER-mediated death in stressed dDCNs. However, 6OHDA triggered caspase-8 activation but this was achieved through a different route and not via the PERK pathway (Figure 6B,C).

## 4. Discussion

Our experimental data from the PD model and the bioinformatics analysis of SARS-CoV-2 transcriptomics shows activation of the ER stress pathway following oxidative stress in dDCNs and in infected cell lines. The development of misfolded proteins due to oxidative stress leading to ER stress is a common feature observed in both viral infections including SARS-CoV-2 and neurodegenerative disorders such as PD [3,46]. Accumulation of these misfolded proteins eventually lead to ER stress and activation of the PERK pathway [47]. Our results, using gene set enrichment analysis, showed that oxidative stress related genes were enriched in response to COVID-19 infection in iPSC-cardiomyocytes and iPSC-type II alveolar epithelial-like cells. Further analysis revealed an upregulation of the antioxidant genes downstream of Nrf2 in all studied cell lines. This is in accordance with other in silico work on inflammatory cells that confirms the upregulation of oxidative stress markers [48]. Moreover, clinical studies have shown that systemic oxidative stress has been associated with severely ill COVID-19 patients who were hospitalized in the intensive care unit [49]. The association of oxidative stress with severity could be attributed to the elevated neutrophil/lymphocyte ratio as the activated neutrophil is the most critical source of the ROS released in COVID-19 infection [50].

**Figure 7:**
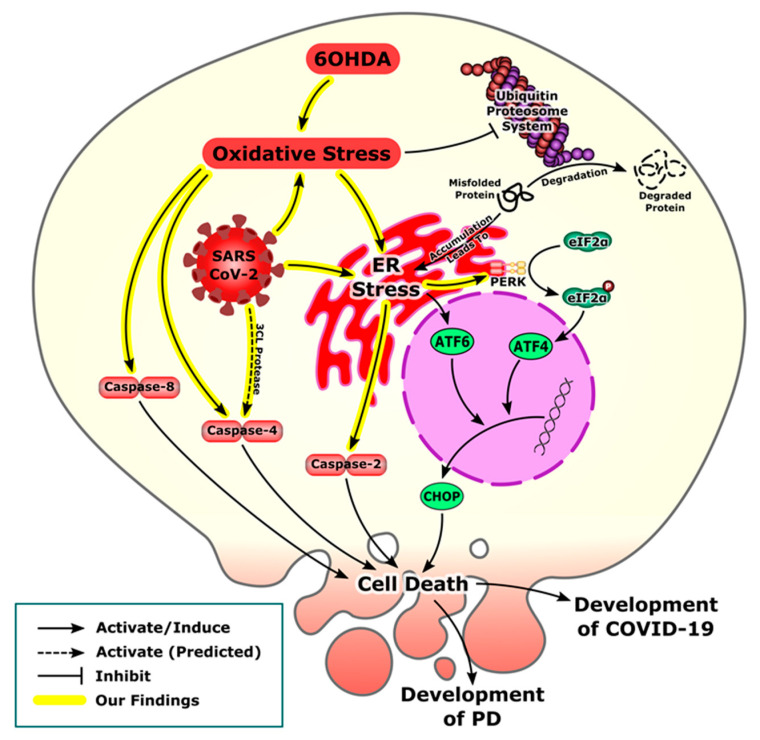
A schematic representation of similarities in cellular events demonstrating activation of cell death mechanisms following oxidative stress leading to the development of PD and COVID-19. A closer examination of our laboratory results, gene set enrichment analysis and available literature suggested that SARS-CoV-2 and 6OHDA triggered oxidative stress followed by stimulation of the PERK, ATF-6 and IRE-1 pathways results in the activation of ER stress and eventually ER-mediated cell death. In addition, 6OHDA-induced oxidative stress caused activation of caspases-2, -4 and-8 using diverse pathways. While 3CL protease, a SARS-CoV-2 protease is predicted to cleave and activate caspase-4.

It is well established that 6OHDA induces neurotoxicity onto the nigrostriatal dopaminergic pathway by producing oxidative stress due to increased production of ROS. In addition, it inhibits the mitochondrial electron chain complexes -I and -IV leading to impairment of mitochondrial membrane potential, which in turn affects ATP production, provoking neuronal degeneration and progression of PD [51,52,53,54,55,56]. It should be noted that our study uses differentiated neurons and that the ER stress-associated events in these cells may differ to those found in PD pathology. The development of oxidative stress either from COVID-19 infection or 6OHDA-induced neurotoxicity can eventually lead to ER stress and the development of the UPR. However, under excessive cellular stress, UPR is extended and activated PERK promotes the phosphorylation and inactivation of the α subunit of eukaryotic initiation factor 2 (eIF2), preventing further translation of new proteins, this helps to reduce the amount of proteins accumulating in the ER and to support cell survival [57]. It is known that salubrinal inhibits eIF2-alpha dephosphorylation, thus enhancing *p*-eIF2-alpha phosphorylation [58]. In addition, PERK promotes translation of activating transcription factor 4 (ATF-4) through the phosphorylation of eIF-2. Subsequently, ATF-4 enters the nucleus and stimulates the transcription of genes which restore ER homeostasis [13,14,16]. The inhibition of more proteins being translated acts as a signal and increases the activity of the transcription factor ATF4 which triggers activation of a proapoptotic C/EBP homologous protein (CHOP) involved in the development of ER stress-induced apoptosis and microbial infection [59,60] (Figure 7).

Gene set enrichment analysis showed that the UPR markers were elevated in the iPSC-Cardiomyocytes infected with COVID-19. Further analysis revealed the activation of the PERK pathway in response to COVID-19 infection not only in iPSC-cardiomyocytes but also in iPSC-neural cells (Figure 2). In addition to the role exerted by the oxidative stress to induce ER stress, the biosynthesis of SARS-CoV-2 virions has a great impact in triggering ER stress. One of the integral structural proteins-envelope (E) protein in SARS-CoV-2 forms hydrophilic pores in endoplasmic reticulum membranes which act as Ca^2+^ ions channel creating disturbances in Ca^2+^ ions homeostasis [61]. Another suggested mechanism of ER stress is that SARS-CoV-2 depletes the ER membrane to form its own envelope, leading to ER stress [3,62].

In the current study, the stimulation of PERK, leading to caspase-2 activity, may have been due to an increased level of ROS caused by 6OHDA-induced toxicity. The accumulation of ROS may possibly have caused misfolding and impairment of proteins leading to initiation of the PERK ER stress pathway followed by apoptotic death of dDCNs (Figure 3). These findings are comparable with the previously published work by Arduíno et al. [30], that demonstrated activation of caspase-2 in tunicamycin treated neuronal cells and revealed that PERK is an upstream protein which promotes activation of caspase-2 in NT2 cells. Our results demonstrated that the suppression of PERK protein can inhibit caspase-2 activation, resulting in an increase in survival of 6OHDA-treated dDCNs (Figure 3). Previous research by Jiang et al. [63] showed that salubrinal reduced caspase-3 activity and protected cells from ER stress-mediated apoptosis in sodium butyrate--treated cells. In comparison, the current study shows that salubrinal suppresses caspase-2 activity leading to an increase in cell survival and slowing death of 6OHDA-treated dDCNs. Collectively, our results demonstrate the similarities between 6OHDA-induced toxicity in dDCNs and SARS-CoV-2 infection regarding the development of oxidative stress that triggers the PERK pathway resulting in ER stress-mediated death of affected dDCNs and/or in cell lines (Figure 7).

**Figure 8:**
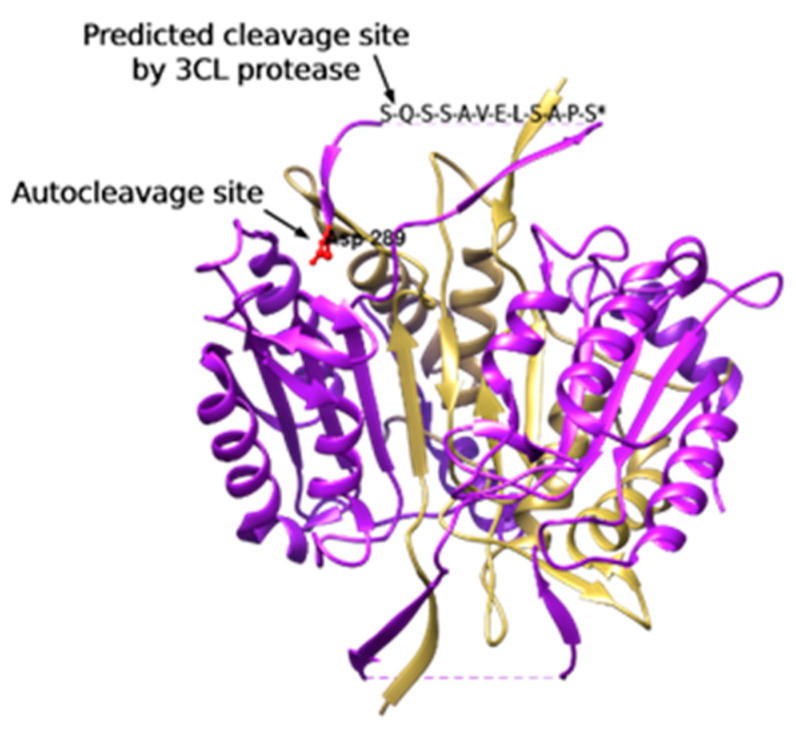
A 3D representation of caspase-4 homodimer showing auto cleavage site at Asp289 and the predicted cleavage site by 3CL protease (Gln281). * Missed loop in the original crystallographic structure has been manually inserted.

The activation of caspases has been shown to mediate apoptosis that can occur via intrinsic or extrinsic pathways. Casapse-4 is localised in ER and is a member of a group of pro-inflammatory caspases along with caspases-1, -5, -11 and -12 [64]. Inflammatory caspases are involved in the activation of inflammatory cytokines as a response to microbial pathogens. Caspase-4 has been shown to regulate activation of non-canonical inflammasomes against bacterial pathogens [65]. Furthermore, a study from Scollberger 2012 [66] supports caspase-4 involvement in the activation of inflammasomes via caspase-1activation. Recently, caspase-4 activation, measured by gasdermin D processing, was proved to be dependent on one of the cleavage events at Asp289 [67]. Surprisingly, that cleavage site is only 8 residues away from the predicted cleavage site by the SARS-CoV-2 3CL protease (Gln281) (Figure 8). As the predicted cleavage site is near the original site that is associated with caspase-4 activation, it is more likely to be also associated with caspase-4 activation. These findings suggest that the predicted cleavage of caspase-4 by 3CL protease may be associated with its activation and perhaps linked with SARS-CoV2 induced stress [67]. These in silico predictions need to be validated, if 3CL protease does cleaves caspase-4 at Gln281 or not, then the activity of the cleaved caspase-4 should be assessed. Similarly in our PD model, caspase-4 specific inhibitor study revealed activation of caspase-4 pathway causing death of 6OHDA-treated dDCNs. Again, we shall highlight the similarities between activation of caspase-4 in 6OHDA-treated dDCNs and COVID-19-predicted activation of the same caspase. In addition, a surprising outcome was achieved in the current study which illustrated the lack of relation of caspase- 4 in PERK ER-mediated death of stressed dDCNs. Our results thus illustrate that salubrinal was unable to suppress caspase-4 activation in 6OHDA-treated dDCNs. Stimulation of caspase-4 activity was found to be acting independently from the PERK pathway in 6OHDA-treated dDCNs (Figure 5). These findings are in contrast to the research carried out by Lee et al. [68] that showed increased levels of caspases-3 and -4 in tunicamycin treated SK-N-SH cells, indicated that caspase-4 was triggered by PERK pathway. Furthermore, salubrinal had reduced caspase-4 activity in tunicamycin treated SK-N-SH cells. On the otherhand, our results show that the PERK pathway did not influence caspase-4 activity in dDCNs. It could be simply due to the different type of neurotoxin used to induce oxidative stress in our model. 6OHDA may have triggered caspase-4 pathway through calcium mechanisms instead of PERK pathway in dDCNs. This potential idea can be supported by research performed by Arduíno et al. [30] which had shown that MPTP treatment caused leakage of calcium from the ER into the mitochondria and initiated activation of caspases-2,-3,-4 and -9 resulting in death of neuronal PD cells. Similarly, research by Higuchi et al. [69] has shown that calcium promoted caspase-4 and calpain activation in transgenic mice that had been injected with kainic acid. Immunofluorescent analysis revealed degeneration of axons and dendrites along with up regulation of caspase and calpain activation signifying that elevated calcium levels provoke calpain and caspase stimulation resulting in degeneration of axons and dendrites. Although no work has been carried out in calpain and caspase activation in dDCNs, it is plausible that calcium imbalance and calpain proteins interact and effect caspase activation and lead dDCNs towards death pathway. It is also possible that in stressed dDCNs, 6OHDA may have triggered caspase-4 activation through a calcium related pathway.

The current study is the first to determine if caspase-8 is involved in ER-mediated death of dDCNs following oxidative stress induced by 6OHDA. Immunocytochemical and WB analysis illustrated caspase-8 activation in presence of the salubrinal, signifying that stimulation of caspase-8 is achieved through a different pathway and is not connected to the PERK pathway in 6OHDA-treated dDCNs. A closer examination of caspase-8 activation in relevance to the PERK pathway in 6OHDA-treated dDCNs revealed that suppressing the PERK pathway and inhibiting caspase-8 pathway promoted further survival of dDCNs (Figure 4). A study by Smith et al. [70] found that caspase-12 participates in ER stress-mediated death of DCNs. Salubrinal partially inhibited the death of DCNs by suppression of caspase-12, indicating that ER stress caused death of DCNs but other pathways such as the mitochondrial pathway also contributed to death of DCNs. In comparison to work carried out by Smith et al. [70], caspase-12 was not detected in the current study (data not shown) instead, caspase-4 was found to be active in 6OHDA-treated dDCNs (Figure 5). A possible reason could be because the human cell has large deletions of essential homologues of caspase-12, whilst in the animal model all the essential codons to promote caspase-12 protein are expressed, meaning that caspase-12 is usually actively expressed in PD animal model but not in the human PD model [70]. Similarly to Smith et al. [70], our results demonstrate that ER stress causes death of 6OHDA-treated dDCNs. In addition, our results demonstrate that suppression of the PERK pathway along with caspases-4 and -8 can promote further survival of dDCNs, indicating the involvement of other pathways as well. In addition, we predict that caspase-4 plays an independent role in COVID-19-induced cell death. As illustrated by the gene set enrichment analysis, COVID-19 susceptible cells suffer from activation of cell death mechanisms that eventually would lead to multiorgan failure. Although COVID-19-induced heart injury could result indirectly from the cytokine storm and increased coagulopathy [71], we found that direct infection of cardiac cells would activate oxidative and ER stress pathways leading to activation of cell death mechanisms. Furthermore, COVID-19-induced nervous manifestations could be divided into direct and indirect effects [72]. We found that the direct effects caused by viral replication in the iPSC-neural cells activated the PERK-ER stress pathway are specifically out of the UPR. Together, these data support the multi-organ failure that has been observed in patients with COVID-19 infections [72] and the crosstalk between PD and COVID-19 that affects neural cells by activating similar cell death mechanisms (Figure 8).

## 5. Conclusions

In summary, our data shows activation of ER stress pathway in the experimental PD model and with bioinformatics analysis of SARS-CoV-2 suggesting that activation of ER stress pathway is a common feature observed in the pathogenesis of both PD and COVID-19. Moreover, suppression of PERK-ER stress pathway had resulted in a significant amount of survival of dDCNs, indicating that 6OHDA-induced oxidative stress provoked PERK-ER-mediated cell death in dDCNs. Further research is needed to elaborate new specific strategies in identifying other key proteins in the PERK-ER stress pathway that may aid in providing effective targets to slow death processes and provide protection to stressed cells.

## Figures and Tables

**Table 1:** NetCorona-1.0 server output: Residue position is the position of the glutamine residue in the caspase-4 protein sequence. Th sequence represents the 10 residues surrounding the candidate cleavage site. The cleavage score is the value assigned by NetCorona-1.0 server to each candidate cleavage site. Cleavage scores greater than 0.5 (NetCorona-1.0 threshold) are marked with *.

Residue Position	Sequence	Cleavage Score
32	NLVEQ^NVLNW	0.088
62	ADSMQ^EKQRM	0.107
65	MQEKQ^RMAGQ	0.062
70	RMAGQ^MLLQT	0.074
74	QMLLQ^TFFNI	0.091
81	FNIDQ^ISPNK	0.071
236	DTIFQ^IFNNR	0.139
256	VIIVQ^ACRGA	0.112
281	VASSQ^SSENL	0.630 *
325	IFITQ^LITCF	0.061
331	ITCFQ^KYSWC	0.082
347	FRKVQ^QSFET	0.076
348	RKVQQ^SFETP	0.379
358	RAKAQ^MPTIE	0.095

## Data Availability

Not applicable.

## Supplementary Materials

The following are available online at https://www.mdpi.com/article/10.3390/brainsci12040507/s1, Table S1: Determining the effect of salubrinal and zVADfmk in stressed dDCNs; Table S2: Determining if caspase-2 is active in ER stress pathway in 6OHDA-treated dDCNs; Table S3: Determining the Amount of caspase-2 in Salubrinal + 6OHDA-treated dDCNs; Table S4: Determining if caspase-4 is active in ER stress pathway in 6OHDA-treated dDCNs; Table S5: Determining the amount of caspase-4 in salubrinal-treated 6OHDA dDCNs; Table S6: Determining if caspase-8 is active in ER stress pathway of 6OHDA-treated dDCNs; Table S7: Determining the Amount of Caspase-8 in Salubrinal + 6OHDA-treated dDCNs.
