# Supplementary material for: ER Stress in COVID-19 and Parkinson’s Disease: In Vitro and In Silico Evidences
Source: Brain Sci. 2022 Apr 16;12(4):507. doi: 10.3390/brainsci12040507 (PMC9025812; doi:10.3390/brainsci12040507)
Supplement: Supplementary file 1 [file brainsci-12-00507-s001.zip › brainsci-1629904-supplementary.pdf]

# Supplementary Material

**Table S1.** Determining the effect of salubrinal and zVADfmk in stressed dDCNs.

| | Mean not treated | Mean salubrinal | Mean zVADfmk | Mean 6OHDA | Mean 6OHDA & zVADfmk | Mean 6OHDA & salubrinal | Mean 6OHDA & salubrinal & zVADfmk |
|---|---|---|---|---|---|---|---|
| **Mean of Assay 1** | 1.98 | 1.87 | 1.89 | 0.65 | 1.18 | 1.50 | 1.82 |
| **Mean of Assay 2** | 1.85 | 1.75 | 1.76 | 0.56 | 1.06 | 1.51 | 1.68 |
| **Mean of Assay 3** | 2.05 | 1.94 | 1.99 | 0.61 | 1.08 | 1.55 | 1.84 |
| **Mean of All Assays** | 1.96 | 1.85 | 1.88 | 0.61 | 1.11 | 1.52 | **1.78** |
| **Mean of All Assays %** | **100** | **94** | **96** | **31** | **56** | **78** | **91** |
| **SD** | 0.10 | 0.09 | 0.11 | 0.05 | 0.06 | 0.03 | 0.09 |
| **(n)** | 3.00 | 3.00 | 3.00 | 3.00 | 3.00 | 3.00 | 3.00 |
| **SQR** | 1.73 | 1.73 | 1.73 | 1.73 | 1.73 | 1.73 | 1.73 |
| **SE** | 0.06 | 0.05 | 0.07 | 0.03 | 0.04 | 0.02 | 0.05 |
| **SE %** | 5.74 | 5.47 | 6.59 | 2.67 | 3.58 | 1.68 | 5.07 |
| **T Test** | | | | | **0.001** | **0.00004** | **0.00012** |

**Table S2.** Determining if caspase-2 is active in ER stress pathway in 6OHDA-treated dDCNs.

| | Mean untreated | Meansalubrinal | MeanzVDVA Dfmk | Mean 6OHDA | Mean 6OHDA & salubrinal | Mean 6OHDA & zVDVADfmk | Mean 6OHDA &salubrinal & zVDVADfmk |
|---|---|---|---|---|---|---|---|
| **Mean of Assay 1** | 2.08 | 2.13 | 2.10 | 0.65 | 1.44 | 1.52 | 1.51 |
| **Meanof Assay 2** | 2.18 | 2.19 | 2.14 | 0.55 | 1.27 | 1.39 | 1.52 |
| **Mean of Assay 3** | 2.12 | 2.11 | 2.15 | 0.73 | 1.46 | 1.35 | 1.48 |
| **Meanof All Assays** | 2.13 | 2.14 | 2.13 | 0.64 | 1.39 | 1.42 | 1.50 |
| **Mean of All Assays %** | **100** | **101** | **100** | **30** | **65** | **67** | **71** |
| **SD** | 0.048 | 0.041 | 0.027 | 0.087 | 0.104 | 0.090 | 0.023 |
| **(n)** | 3 | 3 | 3 | 3 | 3 | 3 | 3 |
| **SQR** | 1.73 | 1.73 | 1.73 | 1.73 | 1.73 | 1.73 | 1.73 |
| **SE** | 0.028 | 0.023 | 0.016 | 0.050 | 0.060 | 0.052 | 0.013 |
| **SE %** | 2.755 | 2.348 | 1.557 | 5.031 | 6.026 | 5.214 | 1.313 |
| **T Test** | | | | | **0.001** | **0.0004** | **0.002** |
| | | | | | | **0.716** | |
| | | | | | | **0.199** | **0.257** |

**Table S3.** : Determining the Amount of caspase-2 in Salubrinal + 6OHDA-treated dDCNs.

| | Mean utreated | Mean salubrinal | Mean 6OHDA | Mean 6OHDA &salubrinal |
|---|---|---|---|---|
| **Caspase-2 Assay 1** | 1.069 | 0.000 | 1.702 | 0 |
| **Caspase-2 Assay 2** | 1.117 | 0.000 | 1.604 | 0 |
| **Caspase-2 Assay 3** | 0.959 | 0.000 | 1.746 | 0 |
| **Caspase-2 Assay 4** | 0.529 | 0.000 | 1.529 | 0 |
| **Caspase-2 Assay 5** | 0.421 | 0.000 | 2.416 | 0 |
| **Mean of All Assays** | 0.819 | 0.000 | 1.799 | 0.000 |
| **Mean of All Assays %** | **100** | **0** | **220** | **0** |
| **SD** | 0.321 | 0.000 | 0.355 | 0.000 |

| | | | | |
|---|---|---|---|---|
| SD (%) | 32.13 | 0.00 | 35.48 | 0.00 |
| T Test | | | 0.02 | |

**Table S4.** Determining if caspase-4 is active in ER stress pathway in 6OHDA-treated dDCNs.

| | Mean un treated | Mean salubrinal | Mean zLEVDfmk | Mean 6OHDA | Mean 6OHDA & salubrinal | Mean 6OHDA & zLEVDfmk | Mean 6OHDA & salubrinal & zLEVDfmk |
|---|---|---|---|---|---|---|---|
| Mean of Assay 1 | 2.03 | 2.02 | 2.10 | 0.72 | 1.43 | 1.22 | 1.57 |
| Mean of Assay 2 | 2.03 | 2.07 | 2.11 | 0.67 | 1.38 | 1.26 | 1.61 |
| Mean of Assay 3 | 2.04 | 2.05 | 2.06 | 0.60 | 1.36 | 1.17 | 1.54 |
| Mean ofAll Assays | 2.03 | 2.05 | 2.09 | 0.66 | 1.39 | 1.22 | 1.57 |
| Mean ofAll Assays % | 100 | 101 | 103 | 33 | 68 | 60 | 77 |
| SD | 0.010 | 0.025 | 0.025 | 0.059 | 0.036 | 0.045 | 0.036 |
| (n) | 3 | 3 | 3 | 3 | 3 | 3 | 3 |
| SQR | 1.73 | 1.73 | 1.73 | 1.73 | 1.73 | 1.73 | 1.73 |
| SE | 0.006 | 0.014 | 0.015 | 0.034 | 0.021 | 0.026 | 0.021 |
| SE % | 0.585 | 1.437 | 1.461 | 3.391 | 2.101 | 2.578 | 2.057 |
| T Test | | | | | 0.0002 | 0.0003 | 0.0001 |
| | | | | | 0.007 | | |
| | | | | | | 0.004 | 0.001 |

**Table S5.** Determining the amount of caspase-4 in salubrinal-treated 6OHDA dDCNs.

| | Mean untreated | Mean salubrinal | Mean 6OHDA | MEAN 6OHDA & SALUBRINAL |
|---|---|---|---|---|
| Caspase-4 Assay 1 | 1.117 | 1.100 | 1.520 | 0.978 |
| Caspase-4 Assay 2 | 0.942 | 0.965 | 1.356 | 1.057 |
| Caspase-4 Assay 3 | 1.164 | 1.134 | 1.468 | 1.576 |
| Caspase-4 Assay 4 | 0.586 | 1.637 | 2.545 | 3.223 |
| Caspase-4 Assay 5 | 1.117 | 0.657 | 1.348 | 1.295 |
| Mean of All Assays | 0.985 | 1.099 | 1.647 | 1.626 |
| Mean of All Assays % | 100 | 112 | 167 | 165 |
| SD | 0.239 | 0.355 | 0.507 | 0.325 |
| SD (%) | 23.89 | 35.50 | 50.71 | 32.51 |
| T Test | | | 0.04 | 0.965 |

**Table S6.** Determining if caspase-8 is active in ER stress pathway of 6OHDA-treated dDCNs.

| | Mean Untreated | Mean salubrinal | Mean zIETD fmk | Mean6OHDA | Mean 6OHDA & zIETDfmk | Mean 6OHDA & salubrinal | MeanN 6OHDA &salubrinal & zIETDfmk |
|---|---|---|---|---|---|---|---|
| Mean of Assay 1 | 2.14 | 2.14 | 2.15 | 0.67 | 1.48 | 1.26 | 1.76 |
| Mean ofAssay 2 | 2.16 | 2.12 | 2.12 | 0.67 | 1.51 | 1.32 | 1.78 |
| Mean of Assay 3 | 1.99 | 2.04 | 2.10 | 0.67 | 1.57 | 1.36 | 1.83 |
| Mean ofAll Assays | 2.10 | 2.10 | 2.12 | 0.67 | 1.52 | 1.31 | 1.79 |
| Mean ofAll Assays % | 100 | 100 | 101 | 32 | 72 | 63 | 85 |
| SD | 0.09 | 0.05 | 0.02 | 0.00 | 0.05 | 0.05 | 0.04 |
| (n) | 3 | 3 | 3 | 3 | 3 | 3 | 3 |
| SQR | 1.73 | 1.73 | 1.73 | 1.73 | 1.73 | 1.73 | 1.73 |
| SE | 0.05 | 0.03 | 0.01 | 0.00 | 0.03 | 0.03 | 0.02 |

| | | | | | | | |
|---|---|---|---|---|---|---|---|
| **SE %** | 5.39 | 2.78 | 1.35 | 0.20 | 2.74 | 3.00 | 2.10 |
| **T Test** | | | | | **0.001** | **0.002** | **0.0003** |
| | | | | | **0.007** | | |
| | | | | | | **0.002** | **0.0004** |

**Table S7.** Determining the Amount of Caspase-8 in Salubrinal + 6OHDA-treated dDCNs.

| | **Mean untreated** | **Mean salubrinal** | **Mean 6OHDA** | **Mean 6OHDA &salubrinal** |
|---|---|---|---|---|
| **Caspase-8 Assay 1** | 0.970 | 0.945 | 1.876 | 1.359 |
| **Caspase-8Assay 2** | 0.980 | 1.082 | 1.336 | 2.154 |
| **Caspase-8 Assay 3** | 0.912 | 0.968 | 1.473 | 1.520 |
| **Caspase-8Assay 4** | 0.871 | 0.830 | 1.954 | 2.373 |
| **Active Caspase-8 Assay 5** | 0.974 | 1.485 | 1.861 | 1.543 |
| **Mean of AllAssays** | 0.941 | 1.062 | 1.700 | 1.790 |
| **Mean of All Assays %** | **100** | **113** | **181** | **190** |
| **SD** | 0.048 | 0.253 | 0.276 | 0.445 |
| **SD (%)** | 4.79 | 25.28 | 27.63 | 44.49 |
| **T Test** | | | **0.003** | **0.713** |
